# Supplementary material for: Corn Yield Prediction With Ensemble CNN-DNN
Source: Front Plant Sci. 2021 Aug 2;12:709008. doi: 10.3389/fpls.2021.709008 (PMC8364956; doi:10.3389/fpls.2021.709008)
Supplement: Supplementary file 1 [file Data_Sheet_1.docx]

**Supplementary information to:**

**Corn Yield Prediction with Ensemble CNN-DNN**

Mohsen Shahhosseini^1^, Guiping Hu^1*^, Saeed Khaki^1^, Sotirios V. Archontoulis^2^

^1^ Department of Industrial and Manufacturing Systems Engineering, Iowa State University, Ames, Iowa, USA

^2^ Department of Agronomy, Iowa State University, Ames, Iowa, USA

* Corresponding author: E-mail: [gphu@iastate.edu](mailto:gphu@iastate.edu)

**Test year: 2018**

**Test year: 2017**

**Test year: 2019**


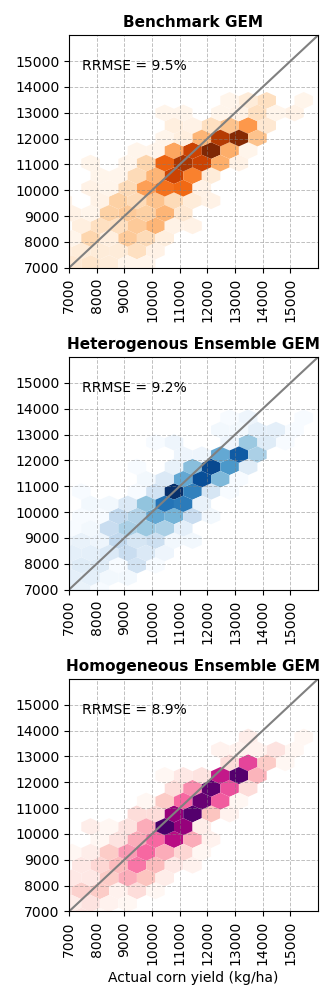

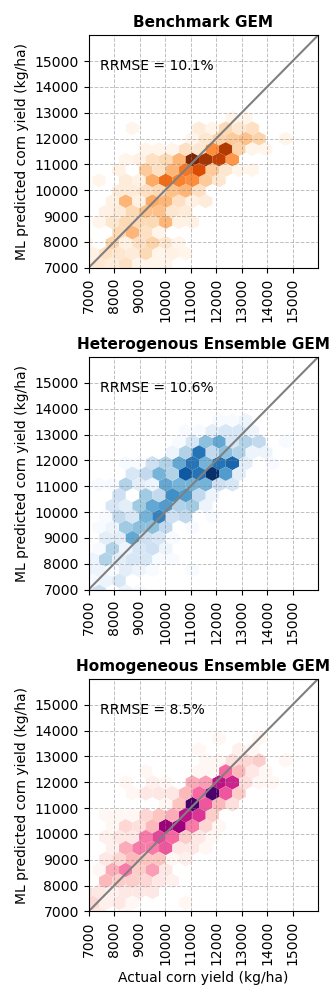

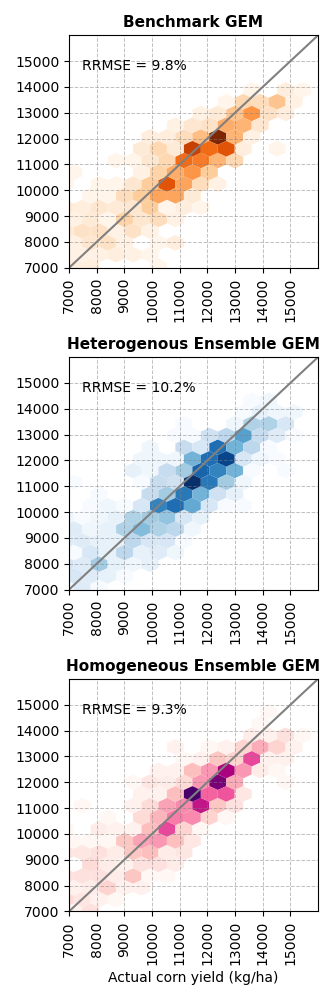


Figure S1: X–Y plots of the created ensemble models and the benchmark ensemble for the test years 2017, 2018, and 2019. The intensity of the colors shows the accumulation of the data points.
